# Circadian rhythm in idiopathic normal pressure hydrocephalus

**DOI:** 10.1186/2045-8118-12-S1-P13

**Published:** 2015-09-18

**Authors:** Andreas Eleftheriou, Martin Ulander, Fredrik Lundin

**Affiliations:** 1 Department of Neurology, University Hospital, Linköping, Sweden; 2 Department of Clinical Neurophysiology, University Hospital, Linköping, Linköping, Sweden; 3 Department of Clinical and Experimental Medicine (IKE), Linköping University, Sweden

## Introduction

The pathogenesis of idiopathic normal pressure hydrocephalus (iNPH) may take place in structures close to the cerebral ventricular system. Suprachiasmatic nucleus (SCN), situated close to the third ventricle, is involved in circadian rhythm and is therefore of interest to study. One of the main symptoms of iNPH is cognitive impairment. Diurnal disturbances are a well-known phenomenon of patients with dementia. Diurnal rhythm has never been studied in iNPH. The aim was to study any changes of the diurnal rhythm and diurnal activity amplitude, before and after shunt operation in iNPH-patients.

## Methods

Twenty consecutive iNPH patients fulfilling the criteria of American iNPH guidelines from 2005, 9 males and 11 females, mean age 73 (49-81) years were included. The patients underwent a pre-operative clinical work-up including 10 meters walk time (w10mt) steps (w10ms), TUG-time (TUGt) and steps (TUGs) and for cognitive function an MMSE score was measured. In order to receive circadian rhythm data actigraphic recordings were performed using the SenseWear 2 (BodyMedia Inc Pittsburgh, PA, USA) actigraph. Cosinor analyses of accelerometry data were performed in “R” using non-linear regression with Levenburg-Marquardt estimation. Pre- and post-operative data regarding mesor, amplitude and circadian period were compared using Wilcoxon-Mann-Whitney test for paired data.

## Results

Twenty patients were evaluated before and three month post-operatively. Motor function (w10mt, w10ms, TUGt, TUGs) was significantly improved while MMSE was not significantly changed (pre: 26/30; post: 27/30). Actigraphic measurements (mesor, amplitude and circadian period) showed no significant changes after the shunt operation.

## Conclusion

This is the first systematic study of circadian rhythm in iNPH-patients. There were no significant changes in circadian rhythm after shunt surgery. This is in line with the results from our previous actigraphic study showing that the patients did not improve their ambulatory activity in spite of improved motor function. Cognition measured with MMSE remained unchanged. These findings underscore the need of more active post-operative rehabilitation.

